# Assessing the severity of functional impairment of psychiatric disorders: equipercentile linking the mini-ICF-APP and CGI

**DOI:** 10.1186/s12955-019-1235-5

**Published:** 2019-11-19

**Authors:** Stephan T. Egger, Godehard Weniger, Mario Müller, Julio Bobes, Erich Seifritz, Stefan Vetter

**Affiliations:** 1 Centre for Integrative Psychiatry, Department of Psychiatry, Psychotherapy and Psychosomatics, Psychiatric University Hospital of Zurich, University of Zurich, Lenggstrasse 31, 8032 Zurich, Switzerland; 2 Department of Psychiatry, Faculty of Medicine, University of Oviedo, CIBERSAM, Oviedo, Spain

**Keywords:** Psychiatric disorders, Functionality, Severity, Mini-ICF-APP, CGI, Equipercentile

## Abstract

**Background:**

The assessment of functioning and impairment due to psychiatric illness has been acknowledged to be crucial for research and practice. This led to the development of the mini-ICF-APP, which provides a reliable and time-efficient measure of functioning and impairment. Although its use is increasing, it remains unclear how it reflects severity and how change might be interpreted from a clinical perspective.

**Methods:**

In a clinical sample of 3067 individuals hospitalized for mental health treatment, we used an equipercentile approach to link the mini-ICF-APP with the Clinical Global Impression scale (CGI) at admission and discharge. We linked the mini-ICF-APP sum score to the CGI-S scale and the mini-ICF-APP proportional change between admission and discharge to the CGI-I scale.

**Results:**

The mini-ICF-APP and CGI scales showed a Spearman correlation of 0.50 (*p* < .000). CGI-S: “borderline-ill” corresponded to a mini-ICF-APP score 1–2; “mildly-ill” to 3–7; “moderately-ill” to 8–15; “markedly-ill” to 16–24; “severely-ill” to 25–37; and “extremely-ill” to a score ≥ 38. The Spearman correlation between the percentage change of mini-ICF-APP sum score and the CGI-I was 0.32 (*p* > .000); “no-change” in the CGI-I corresponded to an increase or decrease of 2%; “minimally-improved” to a mini-ICF-APP reduction of 3–30%; “much-improved” to a reduction of 31–63%; “very-much-improved” to a reduction of ≥64% “minimally-worse” to an increase of 3–34% “much-worse” to an increase of 35–67%; and finally “very-much-worse” with an increase of ≥68%.

**Conclusions:**

Our findings improve understanding of the clinical meaning of the mini-ICF-APP sum score and percentage change in patients hospitalized for treatment.

## Background

Mental and substance use disorders contribute substantially to the global burden of disease, being, directly and indirectly, responsible for a significant amount of mortality and morbidity [1]; to a large extent through delayed access to treatment and services [2]. Furthermore, the impact of mental health on disability and incapacity is on the rise [3–5]. Mental disorders are a huge challenge for public health, compounded by the challenges of psychiatric diagnosis itself [1]. In contrast to most other fields of medicine, psychiatric diagnosis remains primarily restricted to subjective symptoms and observable signs [6]; furthermore, there is a poor correspondence between impairment and symptom load [7–10]. This has led to ongoing controversy about the usefulness of psychiatric diagnoses, as well as their reliability and validity [1, 10, 11].

Accordingly, the WHO (World Health Organisation) introduced the ICF (International Classification of Functionality), designed to assess the particular mix of strengths, weaknesses, and other circumstances which may affect the course of a patient’s illness and treatment response, independent of actual symptom levels [12]. However, the ICF is far too complex and time-consuming to be implemented in everyday clinical practice [7, 13]. The mini-ICF-APP was developed in accordance to the WHO-ICF parameters; it is a 13-item short observer-rating instrument to assess functioning and impairment in persons suffering from mental health problems, irrespective of diagnosis [13].

The original version, as well as the first studies involving the mini-ICF-APP were validated on patients with a non-psychotic mental disorder hospitalized for rehabilitation treatment [13–15]. The subsequent studies of the English and Italian version of the scale covered a broader diagnostic spectrum, including patients with a psychotic disorder [16–18]. Recent studies focused on patients with a chronic mental disorder [19] as well as with posttraumatic stress disorder [20]. Since its introduction, the mini-ICF-APP has been progressively implemented by health care providers, insurance companies and pension funds to assess disability and work impairment [16–18, 21, 22].

The evaluation of functioning and disability has vast implications for those affected since it may impact access to services and financial support (e.g., disability pension). Despite a number of validation studies [13, 16–18], there is limited support for the clinical usefulness and interpretation of the mini-ICF-APP. Although higher scores imply greater severity, its clinical relevance is not fully understood. The Clinical Global Impression Scales (CGI), in contrast, describes the overall clinical impression regarding severity or improvement [23]. Therefore, the CGI has been previously used to establish the usefulness of other clinical measures [24–26]. This study aims to provide clinically meaningful and valid scores for the mini ICF which correspond statistically to CGI levels in a sample of patients hospitalized for treatment.

## Methods

### Sample and procedure

The Centre for Integrative Psychiatry, as part of the Psychiatric University Hospital of Zurich, offers a specialized psychiatric and psychotherapeutic treatment program for patients for whom outpatient treatment alone is insufficient to achieve improvement. We used data from a full five-year cohort of consecutive patients hospitalized for treatment (*n* = 3295). Seven percent (6.91% = 228) of the sample excluded due to one or more missing ICF items. Missing data did not differ from the remaining sample regarding sex, age, education, marital status or primary diagnosis (data not shown). The final sample used for subsequent analyses comprised 3067 patients. The data collected is part of the routine clinical care; the competent ethics committee approved the use of this data for further analysis and publication [KEK-ZH BASEC-Nr. 2017–01766].

### Raters and training

Diagnoses were made by a senior psychiatrist according to the ICD-10 diagnostic criteria [27]. Raters were clinicians; psychiatrists, psychiatry residents or clinical psychologists. All raters were trained regularly trained and supervised on the use of the measures employed in this study. All study measures were rated by the same rater, considering the past seven days. Information was obtained through clinical interviews and direct behavioral observation; or provided by nursing staff, social workers and significant others who were directly involved in the treatment process.

### Diagnostic groups

We defined nine diagnostic groups in accordance with ICD-10 classifications [27]: NCD: Neurocognitive Disorders (ICD-10: F0); AUD: Alcohol Use Disorders (ICD-10: F10); SUD: Substance Use Disorders (ICD-10: F11-F19); SPD: Schizophrenia and other psychotic disorders (ICD-10: F2); BPD: Bipolar Disorders (ICD-10: F31); MDD: Major Depressive Disorders (ICD-10: F32 and F33); AXD: Anxiety Disorders (ICD-10: F4X); PD: Personality Disorders (ICD-10: F6); and NDD: Neurodevelopmental Disorders (ICD-10: F7; F8 and F9).

### Measures

The Clinical Global Impression (CGI) scale is a brief, easy to use and pragmatic tool for the assessment of psychiatric illness severity and changes over time, which is intuitively understood and widely used in clinical practice [23, 28–32]. The CGI consists of three subscales: 1. Severity of Illness (CGI-S), 2. Global Improvement (CGI-I), and 3. Efficacy Index (CGI-E); which will not be used [28]. CGI-S and CGI-I have a seven-point Likert scale response format ranging from 1 representing the “healthy subject” to 7 the “extremely ill subject.” The CGI-I ranges from 1 “significant improvement” to 7 “most severe deterioration,” whereby a score of 4 indicates no change [28]. Ratings of CGI-S and CGI-E refer to the past week, ratings of CGI-I to the time elapsed since the first CGI-S assessment. Consequently, CGI-I assessed at the time of discharge from treatment.

### Mini ICF-APP

The International Classification of Functioning, Disability, and Health (ICF) [33] was introduced by the WHO to supplement the primarily symptom-related description of diseases, such as the ICD-10 [27]. The ICF describes and classifies disorders according to the level of functioning, activity/capacity and context, which are directly or indirectly related to restrictions in social participation. The use of the ICF in clinical practice is restricted due to its complexity and time-consuming nature. The mini-ICF-APP [13], was developed as a short observer-rated scale to assess the level of functioning. The mini-ICF-APP consists of thirteen domains of functioning: (1) adherence to regulations and routines, (2) planning and structuring of tasks, (3) flexibility, (4) competency/efficacy, (5) endurance, (6) assertiveness, (7) contact with others, (8) group integration, (9) family and intimate relationships, (10) leisure activities, (11) self-care, (12) mobility and (13) competence to judge and decide. Each item is rated on a five-point Likert scale from 0 (no disability) to 4 (total disability); with anchor definitions for each item provided in the manual. The qualifying of capacities has to be done in reference to a specified context (e.g. work place, work in general, household, etc.). The scale ranges from 0 to 52 points. The mini-ICF-APP is validated and has good psychometric properties [13, 14, 16, 17].

### Statistical analysis

The analysis included only subjects with complete CGI and mini ICF data available at baseline. Simple descriptive statistics were used to represent the demographic and clinical characteristics of the sample. We used a multivariate regression to determine correlation between demographic and clinical variables (gender, age, education and main diagnosis) and the variables used for equipercentile analysis. For the qualitative variables we used the kernel smoothing method for mixed data types. Spearman’s rank coefficient used for pairwise correlations. Equipercentile analysis was used to compare the mini-ICF-APP scale with the CGI scales. In equipercentile linking corresponding cut-points on two different scales are determined, it does this by identifying scores on both measures that have the same percentile rank [34, 35]. In a first step the distribution of both study measures was generated; in a second step distributions were log-linear smoothed, and the corresponding percentiles of both tests matched in the following manner: The CGI-S was linked to the sum score of the mini-ICF-APP; while the CGI-I to the percentage change of the mini-ICF-APP score, between admission and discharge. Anchor values were defined by a CGI-I score of 4 (“no change”) and a mini-ICF-APP percentage change of zero (“0”). Statistical analyses conducted by the statistical software R (v3.5.1), for multivariate regression we used the package “np” (v0.60–9); for equipercentile calculation we used the package “equate” (v2.0.7) [34].

## Results

The study population had an age between 16 and 77 years (43.4 ± 11.9 years); 66.16% were male. The majority of the sample was single (53.14%), had started apprenticeship or college/university education (67.53%). Patient admission was primarily voluntary (94.95%) with a mean length of stay of 40.57 ± 32.46 days. Almost all individuals were routinely discharged (73.62%). The most common diagnosis was AUD (53.60%), followed by SPD (15.29%). A summary of the demographic characteristics of the complete sample is in Table 1. At admission the mini ICF sum score was 19.20 ± 9.89; the CGI- S was 5.31 ± 0.89. At discharge the mini-ICF-APP score was 16.04 ± 9.55; the CGI-S score was 4.73 ± 1.16; the CGI-I score 2.75 ± 0.96.

**Table 1:** Subject Demographics and main diagnosis

Variable	
Age in years *mean, (SD)*	43.4 (11.9)
Female *n, (%)*	1038 (33.84)
Civil Status *n, (%)*	
Single	1691(55.14)
Married	512 (16.70)
Separated	128 (4.17)
Divorced	677 (22.07)
Widowed	54 (1.76)
Unknown	5 (0.16)
Education *n, (%)*	
Primary School	777 (25.33)
Secondary School	98 (3.19)
Apprenticeship	1714 (55.89)
College/University	357 (11.64)
Unknown	121 (3.95)
Main Diagnosis *n, (%)*	
NCD	45 (1.47)
AUD	1644 (53.60)
SUD	135 (4.40)
SPD	469 (15.29)
BPD	80 (2.61)
MDD	272 (8.87)
AXD	110 (3.59)
PD	276 (9.00)
NDD	36 (1.17)

Legend: *NCD* Neurocognitive Disorders, *AUD* Alcohol Use Disorders, *SUD* Substance Use Disorders (except AUD), *SPD* Schizophrenia and other psychotic disorders, *BPD* Bipolar Disorder, *MDD* Major Depressive Disorder, *AXD* Anxiety Disorder, *PD* Personality Disorders, and *NDD* Neurodevelopmental Disorders

### Multivariate regression

The regression model found a statistic significant correlation between the mini-ICF-APP sum score and the CGI-S score (*p* < 0.001); as well as for the mini-ICF-APP percentage of change and the CGI-I score (p < 0.001). Both correlations were independent of gender; age; diagnosis, education or civil status; with *p* values over the significance threshold of 0.05 for all variable levels. In addition the correlation between percentage of change and improvement was independent from the initial severity.

### Linking CGI-S and the mini-ICF-APP sum score

**Fig. 1:**
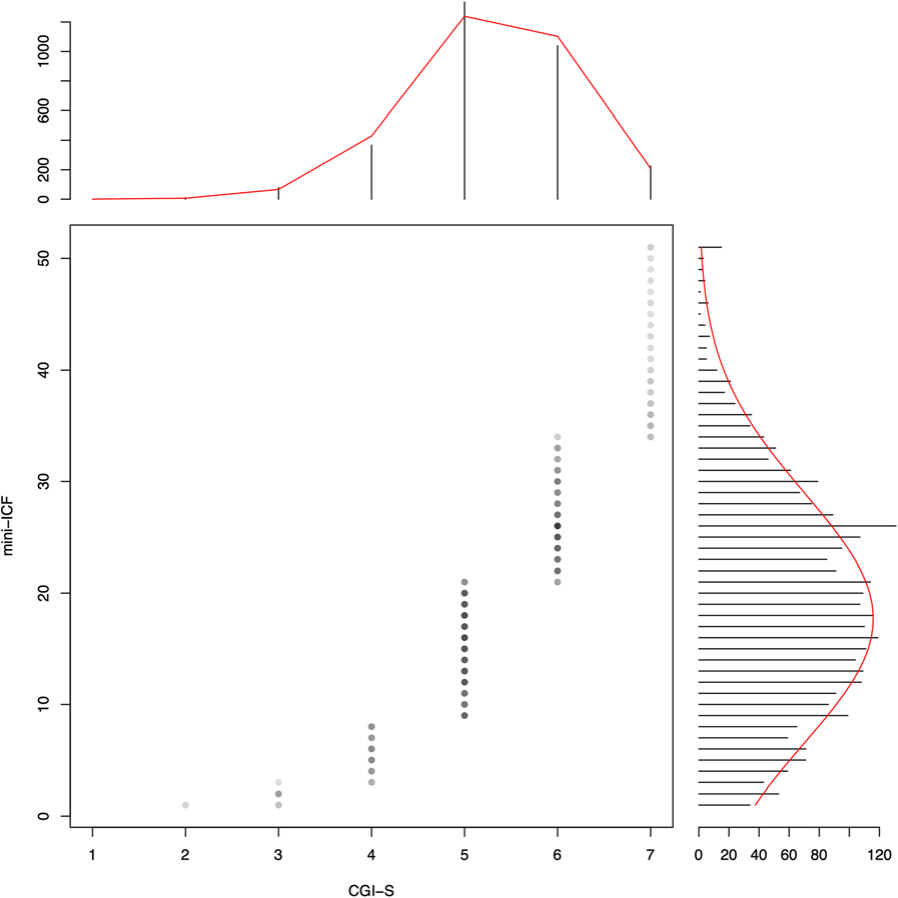
Equipercentile linking of the mini-ICF-APP sum score and the CGI-S. CGI-S values corresponding: 1: “Normal”; 2. “Borderline Ill”; 3. “Mildly Ill”; 4. “Moderately Ill” 5. “Markedly ill”; 6. “Severely ill”; and 7. “Extremely ill”

We found a Spearman correlation coefficient between the CGI-S and the mini-ICF-APP sum score at admission of 0.50 (*p* < .001) for the total sample. Diagnostic groups were found to have significant correlations coefficients (between 0.44 and 0.67); except for NDD (0.16, n.s.). After excluding NDD the total sample correlation was 0.51 (*p* < .001). At discharge, the correlation between the CGI-S and mini-ICF-APP sum score was 0.54 (p < .001). The CGI-S score of 2 (“borderline ill”) corresponded to a mini-ICF-APP score between 1 and 2; CGI-S scores of 3 (“mildly ill”) to a mini-ICF-APP score between 3 and 7; CGI- S scores of 4 (“moderately ill”) to mini-ICF-APP scores between 8 and 15; CGI-S scores of 5 (“markedly ill”) to mini-ICF-APP scores between 16 and 24; CGI-S score of 6 (“severely ill”) to mini-ICF-APP scores between 25 and 37; finally CGI-S score of 7 (“extremely ill”) to a mini-ICF-APP score of 38 or more. Cut-off values are summarized in Table 2 and represented in Fig. 1.

**Table 2:** Cut- off values for the mini-ICF-APP sum score according to CGI-S

CGI- S	mini-ICF-APP sum score
Normal	0–1
Borderline Ill	1–2
Mildly Ill	3–7
Moderately Ill	8–15
Markedly Ill	16–24
Severely Ill	25–37
Extremely Ill	> 38

### Linking CGI-I and the mini-ICF-APP percentage change

The Spearman correlation coefficient between the CGI-I and the mini-ICF-APP sum score difference was 0.30 (*p* < .001); and between the CGI-I and the percentage change of mini-ICF-APP sum score, 0.32 (p < .001). The CGI-I score of 4 (“no change”) was linked to a percentage increase or decrease of 2 percentage points in the mini-ICF-APP; the CGI-I score of 3 (“minimally improved”) to a percentage reduction between 3 and 30 points; the CGI-I score of 2 (“much improved”) to a percentage reduction between 31 and 63 points; the CGI-I score of 1 (“very much improved”) to a percentage reduction of 64 or more; a CGI-I score of 5 (“minimally worse”) to a percentage increase between 3 and 34 points; a CGI-I score of 6 (“much worse”) with a percentage increase between 35 and 67; and finally a CGI-I score of 7 (“very much worse”) with a percentage increase of 68 or more. Cut-off values are summarized in Tables 2 and 3.

**Table 3:** Cut- off values for the percentage change according to the CGI-I

CGI- I	mini-ICF-APP percentage
Very much improved	< 63
Much Improved	(−) 30–62
Minimally Improved	(−) 2–29
No Change	0
Minimally Worse	(+) 2–33
Much Worse	(+) 34–66
Very much worse	> 67

## Discussion

This approach of equipercentile linking allows the definition of severity cut-off values for the mini-ICF-APP according to clinical judgment. Taken together this provides a better understanding and interpretation of the mini-ICF-APP in day-to-day clinical practice. This is of singular importance since the mini-ICF-APP is increasingly being used by health insurance companies and pension funds to determine disability or incapacity benefits caused by a psychiatric condition [21]. Our large clinical sample included diagnoses underrepresented in prior studies with the inclusion of: alcohol and substance use disorders; personality disorders; neurocognitive and neurodevelopmental disorders.

The results of the multivariate analysis did not show a statistical correlation between the mini-ICF-APP and CGI scales and demographic and clinical variables (age, gender, education and main diagnosis). Between the mini-ICF and CGI we were able to show a strong statistical correlation. The correlations found show that illness severity at large is associated with capacity limitations in general. The pairwise comparison by of the scales according to the main diagnosis, produce similar associations between both scales for practically all diagnostic groups. In our analysis age, gender, education and main diagnosis seemingly have no effects on the anchor points between the mini-ICF-APP and CGI scales. Furthermore, change and improvement were independent of the baseline severity. Therefore, we consider that the mini-ICF-APP can be viewed as a universally applicable scale for a diagnosis-independent judgment of functionality; broadening the applicability of the scale.

The mini-ICF-APP severity cut-off points we propose for the mini-ICF-APP are roughly comparable with the values reported in previous studies [16–18, 21, 36]. A direct contrast is however not possible, since the approach to severity assessment differs; with either different gradings of severity [36]; gross allocation of severity grades to mini-ICF-APP values [16, 18]; or the use of psychosocial attributes of severity [17, 21]. Our findings regarding the percentage change are in line with previous findings using the mini-ICF-APP to assess the effectiveness of treatment [14]. Furthermore, our results also concord with the general (although controversially discussed) parameters used to judge improvement [37, 38], were a reduction of 20–30% is considered an indication of response to treatment while a reduction greater than 50% is seen as a significant improvement [39, 40]. The difficulties comparing severity rankings and improvement are not surprising since most available studies dealt with validation issues [13, 16–18].

The severity of a disorder has waste clinical implications, it influences decisions regarding the type and intensity of treatment, whether to continue or stop treatment, but also affect the assess to assistance or disability benefits [41]. The assessment of severity in psychiatric disorders is, however, an issue of ongoing debate, with no resolution insight [41, 42]. The CGI is considered a pragmatic and intuitive scale [23]; that is commonly used to approach severity and change [41, 43–45]. The CGI is considered useful and necessary in routine clinical practice as well as research settings [32]; as well as previously used for the determination of severity cut-off values [24, 26, 46, 47].

Equipercentile linking, allows for a nominal translation from one scale to another, identifying those scores on both scales which have the same percentile ranks [35]. It’s is used in testing programs that involve multiple test forms, like in the educational system [34, 35]. Equipercentyle analysis does not require a specific distribution type and allows for possible measurement errors on both scales compared [35], for this reason, it is considered the preferred linking method amongst other available methods [47, 48]. With an increasingly large number of published studies using this method comparing scales in the fields of psychiatry and neurology [49–52]; with the CGI widely used as a determinant of severity ranking [24–26, 46, 47, 53, 54].

The main strength of our study is the large sample population, without inclusion or exclusion criteria, with a broad diagnostic range, including diagnoses underrepresented in previous studies. The similar correlation coefficients and linking values for the different diagnostic categories confirms the use of the mini-ICF-APP independent of diagnosis [13, 16, 17]. The inclusion of one study site determines some peculiarities in the sample studied. In our case, the large number of patients with AUD is, due to a detoxification treatment program, with a high admission capacity. The over-representation of male patients might be a potential limitation; however, neither the regression model nor the post hoc analysis revealed an effect of gender on the results. One limitation of our study is the recruitment of the sample from patients hospitalized for treatment, which limits the interpretation of the data to these patients requiring treatment. In this context also the influence of the different health systems on treatment seeking behaviour and utilization of mental health services should considered [55].

Our results should be interpreted with caution; since the mini-ICF-APP sum score does not reflect a global incapacity index. Patients with limitations in certain dimensions still could experience impairment; due to the repercussion in other domains. Furthermore, it also should consider how the individual human beings deal with incapacity and seek for solutions on their own. We consider that the CGI scales take account all of these phenomena; with the resulting rather exponential correlation between both scales.

## Conclusions

The results demonstrate in general that illness severity is associated with capacity limitations and that functional change corresponds to a clinical impression of improvement. Our findings contribute to a deeper understanding and clinical interpretation of the mini-ICF-APP.

## Data Availability

The protocol presented and approved by the ethics committee does not foresee to share the data. On reasonable request, the corresponding author will submit an amendment to the ethics committee asking for approval of data sharing in that particular case.

## Abbreviations

AUD: Alcohol Use Disorders
AXD: Anxiety Disorders
BPD: Bipolar Disorders
CGI: Clinical Global Impression Scales
ICD: International Classification of Disease
ICF: International Classification of Functionality
MDD: Major Depressive Disorders
NCD: Neurocognitive Disorders
NDD: Neurodevelopmental Disorders
PD: Personality Disorders
SPD: Schizophrenia and other psychotic disorders
SUD: Substance Use Disorders
WHO: World Health Organisation
